# Supplementary material for: Identification of people with low prevalence diseases in administrative healthcare records: A case study of HIV in British Columbia, Canada
Source: PLoS One. 2023 Aug 31;18(8):e0290777. doi: 10.1371/journal.pone.0290777 (PMC10470893; doi:10.1371/journal.pone.0290777)

**Additional details regarding the validation sub-sample**

**Data quality processing on HIV testing data**

Of persons with positive HIV tests in the BCCDC data linked within the STOP HIV/AIDS data linkage (n=8,219), 675 were excluded by having their earliest positive HIV test date occurring outside the study period. An additional 9 persons were omitted by having a confirmed death date occurring on or before their earliest positive HIV date. For having their latest negative HIV test date (latest before the positive HIV test date) occurring outside the study period (1 April 1996 to 31 March 2020), 307 persons were omitted, while an additional 16 persons were removed by having their negative HIV test date on or after their positive HIV test date. A further 74 persons were omitted by having indications of HIV positivity (receipt of antiretroviral medication dispensations and/or detectable HIV plasma viral loads) before their negative HIV test date. Finally, an additional 14 persons were omitted due to data quality concerns with their death records (e.g., multiple death dates – from different sources – several months or years apart).

**Characterizing the validation sub-sample**

Those included in the validation sub-sample (i.e., those with valid HIV test results from BCCDC) were similar to those in the rest of the STOP HIV/AIDS data linkage (i.e., those without valid HIV test records from BCCDC). The proportion of males was similar – 80.1% for the validation sub-sample vs. 81.5% for rest of the STOP study; the proportion residing in Vancouver Coastal Health Authority (home to BC’s largest municipality: Vancouver) was comparable albeit slightly lower for the validation sub-sample (48.7%) than for the rest of the STOP HIV/AIDS data linkage (54.4%); age at earliest HIV-related record was similar (median: 37 for both subsets). The annual rate of all MSP-billed outpatient healthcare practitioner encounters (for any reason) was similar at a median of 9.7 encounters/year for the validation sub-sample vs 9.9 for the rest of the STOP HIV/AIDS data linkage. Outpatient healthcare practitioner encounters were defined as each unique occurrence of patient/practitioner/date in the MSP Payment Information File, with a service location of A, O, T, P, C, or R (indicating healthcare practitioner office, home, or long-term care settings).

**Table S1. Characteristics of the validation sub-sample and remainder of the STOP HIV/AIDS data linkage**

|  | **Validation**  **sub-sample**  (n=7,124) | **Rest of the STOP HIV/AIDS data linkage**  (n=8,833) |
| --- | --- | --- |
| Male | 80.1% | 81.5% |
| Median (Q1, Q3) age at earliest HIV-related record | 37.0 (30.0, 46.0) | 37.0 (31.0, 44.0) |
| VCHA resident at time of earliest HIV-related record | 48.7% | 54.4% |
| Median (Q1, Q3) number of all-cause outpatient healthcare practitioner encounters per year | 9.7 (9.0, 10.3) | 9.9 (9.5, 10.3) |
| Median (Q1, Q3) years of healthcare records after earliest HIV-related record | 9.2 (3.6, 15.6) | 9.4 (3.2, 19.8) |

*Q1: First quartile; Q3: Third quartile; VCHA: Vancouver Coastal Health Authority. Rest of STOP cohort comprised of persons in the STOP cohort (i.e., persons with an ART dispensation or detectable PvL for HIV, a positive HIV test reported to BCCDC, or an HIV-delated death) who were not included in the validation sub-sample due to lacking valid BCCDC HIV test records.

Additionally, within the validation sub-sample of persons with valid HIV tests – those with solely positive tests (P) were similar to those with both negative and positive tests (N+P) across various characteristics. Similarities were observed in terms of the proportion of males (N+P: 76.7% vs P: 80.6%), the distribution of region of residence at HIV diagnosis – Vancouver Coastal Health Authority (N+P: 51.4% vs P: 49.4%), and the median age at positive HIV test date (N+P: 37 vs P: 38). The median annual rate of all MSP-billed outpatient healthcare encounters was higher for persons with a negative and a positive HIV test (12.2) versus those with a positive HIV test (8.2).

**Table S2. Characteristics of the validation sub-sample, by testing records present**

|  | **Validation**  **sub-sample**  (n=7,124) | | **Remainder of the STOP HIV/AIDS data linkage** (n=8,833) |
| --- | --- | --- | --- |
|  | **Persons with a positive HIV test only**  (n=4,307) | **Persons with a positive and a negative HIV test**  (n=2,817) |  |
| Male | 81.8% | 77.5% | 81.5% |
| Median (Q1,Q3) age at earliest HIV-related record | 38 (31.0, 46.0) | 37 (29.0, 45.0) | 37 (31.0, 44.0) |
| VCHA resident at time of earliest HIV-related record | 47.7% | 50.2% | 54.4% |
| Median (Q1, Q3) number of all-cause outpatient healthcare practitioner encounters per year | 8.2 (7.7, 8.6) | 12.2 (11.1, 12.8) | 9.9 (9.5, 10.3) |
| Median (Q1,Q3) years of healthcare records after earliest HIV-related record | 8.7 (4.0, 13.8) | 9.7 (3.3, 16.8) | 9.4 (3.2, 19.8) |

*Q1: First quartile; Q3: Third quartile; VCHA: Vancouver Coastal Health Authority. Rest of STOP cohort comprised of persons in the STOP cohort who were not included in the validation sub-sample due to lacking valid BCCDC HIV test records.

**Details on the HIV tests reported in the BCCDC testing data**

Since 2009, BC has adopted serological testing algorithms to maximize detection of acute infection, which is disproportionately associated with onward infection. Although the approach has evolved over time, it is premised on using 4^th^ generation dual enzyme immunoassay testing on all seropositive screened individuals and performing HIV RNA in those who are type specific immunoblot indeterminate or negative. Those that are HIV RNA positive and immunoblot indeterminate or negative are reported as presumptive acute infections with a request for serological follow up to document seroconversion. It should be noted that early treatment can delay/blunt immunoblot seroconversions, hence linkage to care and treatment may be necessary to confirm HIV status. In contrast, established HIV infections are typically immunoassay and immunoblot positive at initial testing. The laboratory automatically checks for prior HIV testing history to help differentiate false positives which typically generate low positive immunoassay signals that are maintained with serial testing. Of note, PrEP poses a challenge to serological testing as PrEP is known to blunt both serological as well as HIV RNA detection. This challenge highlights the need for using linked laboratory, clinical, and administrative health data to comprehensively assess disease burden.

Various testing technologies were used for the HIV testing reported in the BCCDC testing file: Antibody, Antigen, and Nucleic Acid Amplification Tests (NAAT). Antibody tests measure antibodies to HIV, proteins that are made by the immune system after the body is exposed to HIV. A confirmatory Assay (an Immunoblot test) is an antibody test, which is used to confirm a preliminary positive HIV test result from a rapid/point-of-care test. Approximately 95% of people with HIV will have antibodies six weeks after exposure, and 99% will have antibodies by three months after exposure (49).

Antigen tests search for an antigen (or viral protein) that is called p24 antigen and appears in the blood of a person shortly after becoming infected with HIV. Specifically, the 4th generation Enzyme Immunoassay Test (EIA) test is a combined antigen/antibody test, and is the standard HIV laboratory screening test used for HIV in BC (49). Traditionally, waiting three months after HIV exposure was recommended, to ensure greater sensitivity of an HIV test however, 4th generation EIA tests have reduced that waiting period: 90% of EIA tests will indicate a positive result by six weeks after a person is exposed to HIV (49).

The NAAT (also known as an RNA test) searches for genetic material of HIV present in blood. The NAAT is the standard test for HIV RNA in BC (50). An estimated 90% of NAATs yield a positive result 10 – 12 days after a person becomes infected with HIV; over 99% of NAATs are positive after six weeks (50). Testing modality also changed over the study period, such that Western Blot was used initially but then replaced by Immunoblot.

**S1 Figure. Visualizations of algorithm search windows**


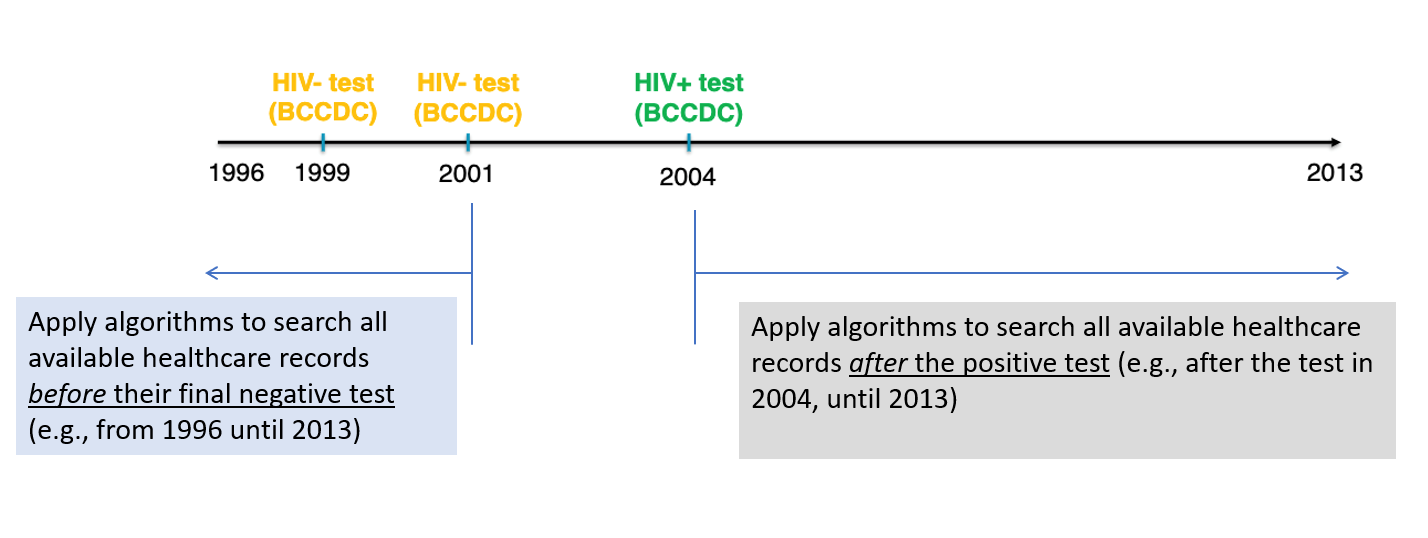


Note that the above year values are fictious (an example of a person with healthcare records from 1996 to 2001 as the period before their last negative HIV test, and with healthcare records from 2004 to 2013 as the period after their first positive HIV test), included for illustrative purposes only.

In the study, to estimate specificity, all available healthcare records were searched before their final negative HIV test date until their first available healthcare record (within the study period: April 1996 to March 2020). Similarly, to estimate sensitivity, all available healthcare records were searched after their first positive HIV test date until their last available healthcare record (within the study period: April 1996 to March 2020).

**S2 Figure. Visualizations of algorithm search windows for sensitivity/specificity**


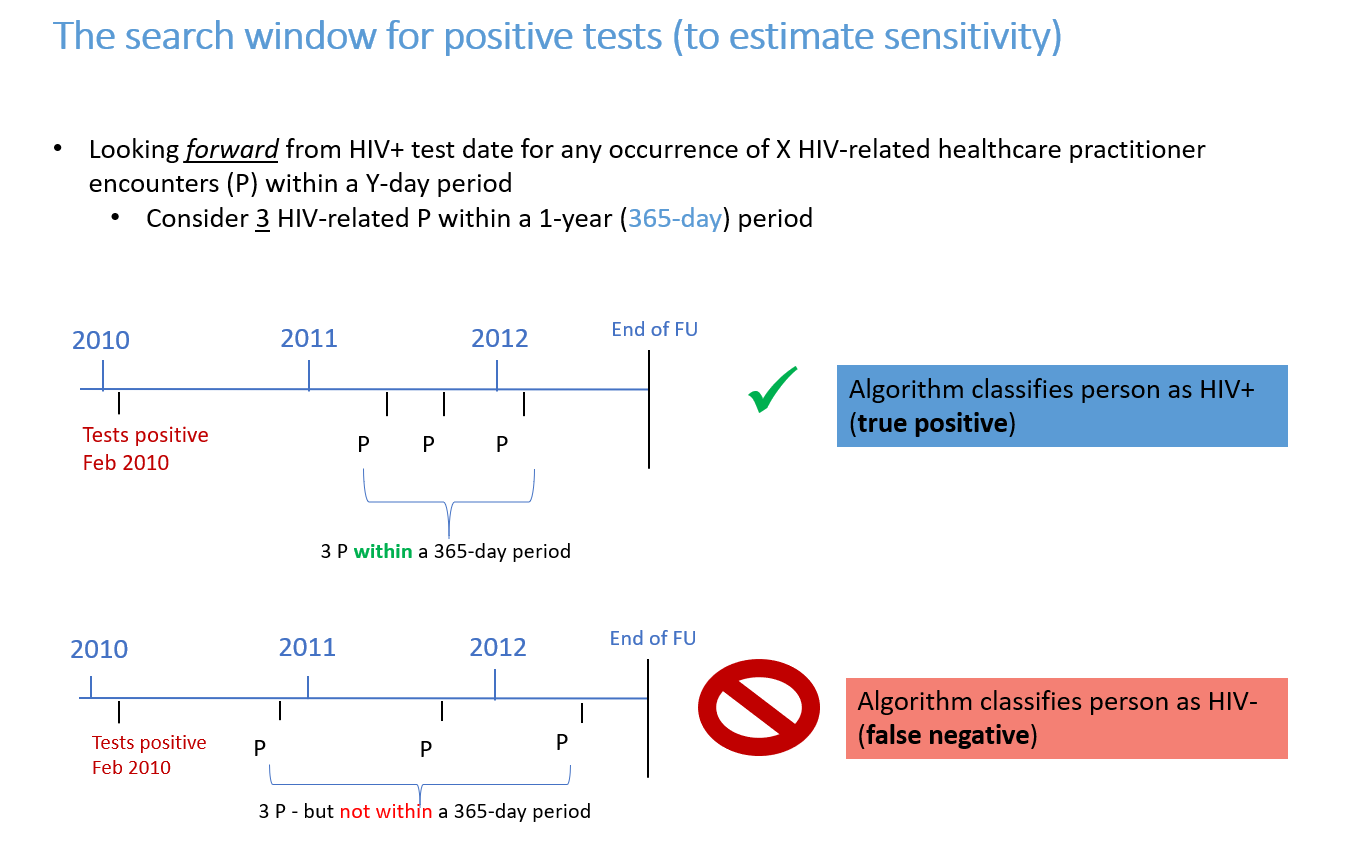


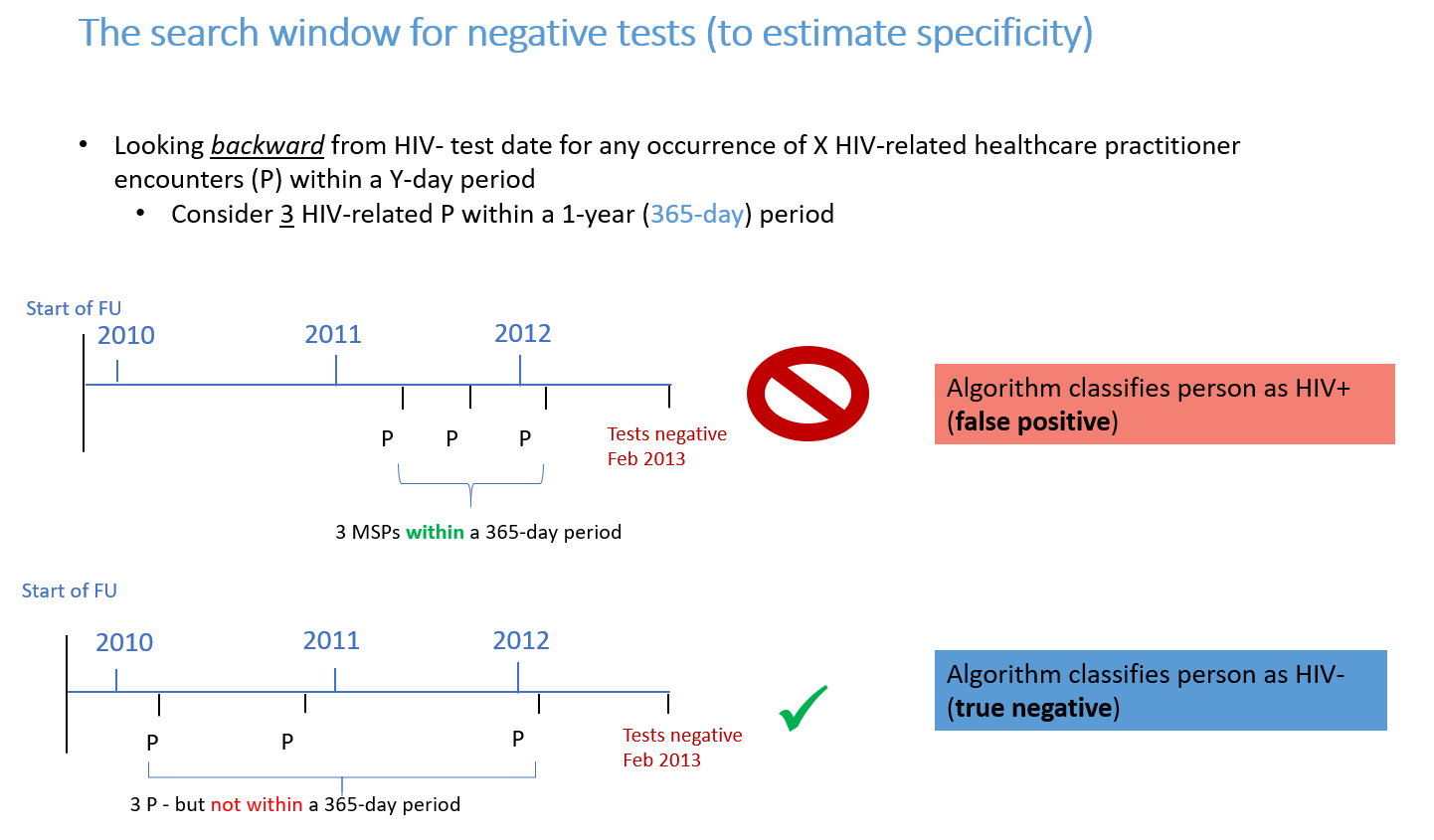


**HIV-related ICD codes for case-finding algorithms**

Due to potential data entry issues when entering ICD diagnostic codes into the MSP and DAD datafiles (e.g., occasionally trailing zeros occur after a legitimate ICD code in MSP records: See: Diagnostic Codes in MSP Claim Data, Summary Report. Medical Services Plan; 1996), we included relevant codes beginning with the codes listed in Table D1 (for healthcare practitioner encounters) and Table D2 (for hospitalizations).

**Table S3. HIV diagnostic codes for identifying HIV-related healthcare practitioner encounters in MSP data**

| **ICD-9** | |
| --- | --- |
| 042x | Human Immunodeficiency Virus (HIV) infection with specified conditions |
| 043x | Human Immunodeficiency Virus (HIV) infection causing other specified conditions |
| 044x | Other Human Immunodeficiency Virus (HIV) infection |
| 795.8x | Positive HIV antibody serology or positive viral culture findings for HIV |
| **ICD-9-CM** | |
| 042x | Human Immunodeficiency Virus (HIV) disease |
| 043x | AIDS-related complex |
| 044x | Other HIV disease |
| 795.71x | Nonspecific serologic evidence of Human Immunodeficiency Virus [HIV] |
| V08x | Asymptomatic HIV infection status |

*Note that ‘x’ indicates that codes beginning with these values were searched. Two ICD-9-CM codes with ‘HIV’ in the description were omitted from our searches: ‘V65.44 Human immunodeficiency virus [HIV] counselling’ was omitted because this code can be used when practitioners are discussing risks related to HIV, rather than solely active management for HIV; ‘079.53 Human immunodeficiency virus [HIV], type 2 [HIV-2]’ was omitted because in the BC context, HIV type 2 is extremely rare and has different disease characteristics, and treatment recommendations than HIV type 1 – which represents virtually all HIV cases in BC (as well as in Canada, more generally) (51).

**Table S4. HIV diagnostic codes for identifying HIV-related hospitalizations in DAD data**

| **ICD-9** | **1 Apr 1996 – 31 Mar 2001** |
| --- | --- |
| 042x | Human Immunodeficiency Virus (HIV) infection with specified conditions |
| 043x | Human Immunodeficiency Virus (HIV) infection causing other specified conditions |
| 044x | Other Human Immunodeficiency Virus (HIV) infection |
| 795.8x | Positive HIV antibody serology or positive viral culture findings for HIV |
| **ICD-9-CM*** | **1 Apr 1996 – 31 Mar 2001** |
| 042x | Human Immunodeficiency Virus (HIV) disease |
| 043x | AIDS-related complex |
| 044x | Other HIV disease |
| 795.71x | Nonspecific serologic evidence of Human Immunodeficiency Virus [HIV] |
| V08x | Asymptomatic HIV infection status |
| **ICD-10-CA** | **1 Apr 2001 – 31 Mar 2020** |
| B24x | Human Immunodeficiency Virus [HIV] disease |
| Z21x | Asymptomatic Human Immunodeficiency Virus [HIV] infection status |
| R75x | Laboratory evidence of Human Immunodeficiency Virus [HIV] |
| O98.7x | HIV disease complicating pregnancy, childbirth and the puerperium |

*Although ICD-9-CM diagnostic codes can and do appear in BC DAD files, it is worthwhile to note that we found no record of 9-CM specific HIV codes (795.71 or V08) in the DAD in the STOP HIV/AIDS study linkage; we list these codes for completeness as it is possible they may appear in queries completed by other researchers. Note that ‘x’ indicates that codes beginning with these values were searched.

‘F02.4 Dementia in human immunodeficiency virus [HIV] disease’ was omitted because this code is required to be entered in conjunction with B24 (which is included in our list), and thus would be redundant if included; ‘Z71.7 Human immunodeficiency virus [HIV] counselling’ was omitted because this code can be used when practitioners are discussing risks related to HIV, rather than solely active management for HIV; ‘Z11.4 Special screening examination for human immunodeficiency virus [HIV]’, ‘Z20.6 Contact with and exposure to human immunodeficiency virus [HIV]’, and ‘Z83.0 Family history of human immunodeficiency virus [HIV] disease’ were each omitted from searches because they do not indicate actively having HIV but related to screening/testing for HIV, exposure to HIV, and having a family history of HIV – respectively.

**Detailed algorithm results (sensitivity, specificity, C-statistic, kappa)**

**Table S5. Specificity estimates (95% confidence intervals), stratified by search window**

|  | **Search window length (time-window in which HIV-related healthcare events must co-occur)** | | |
| --- | --- | --- | --- |
|  | **12-month** | **24-month** | **36-month** |
| **P1H1*** | 97.13%  (96.50%,97.76%) | | |
| **P1H2** | 97.47%  (96.87%,98.06%) | 97.47%  (96.87%,98.06%) | 97.47%  (96.87%,98.06%) |
| **P2H1** | 98.99%  (98.62%,99.37%) | 98.99%  (98.62%,99.37%) | 98.99%  (98.62%,99.37%) |
| **P2H2** | 99.37%  (99.07%,99.67%) | 99.37%  (99.07%,99.67%) | 99.37%  (99.07%,99.67%) |
| **P3H1** | 99.37%  (99.07%,99.67%) | 99.37%  (99.07%,99.67%) | 99.37%  (99.07%,99.67%) |
| **P3H2** | 99.74%  (99.55%,99.93%) | 99.74%  (99.55%,99.93%) | 99.74%  (99.55%,99.93%) |
| **P4H1** | 99.52%  (99.25%,99.78%) | 99.48%  (99.21%,99.75%) | 99.48%  (99.21%,99.75%) |
| **P4H2** | 99.89%  (99.76%,100.00%) | 99.85%  (99.71%,100.00%) | 99.85%  (99.71%,100.00%) |
| **P5H1** | 99.52%  (99.25%,99.78%) | 99.48%  (99.21%,99.75%) | 99.48%  (99.21%,99.75%) |
| **P5H2** | 99.89%  (99.76%,100.00%) | 99.85%  (99.71%,100.00%) | 99.85%  (99.71%,100.00%) |

*The H1 and P1 events were unbounded by search windows since they contained single events; hence, the algorithm P1H1 referred to 1 HIV-related healthcare practitioner, or 1 HIV-related hospitalization occurring at any time.

**Table S6. Sensitivity estimates (95% confidence intervals), stratified by search window**

|  | **Search window length (time-window in which HIV-related healthcare events must co-occur)** | | |
| --- | --- | --- | --- |
|  | **12-month** | **24-month** | **36-month** |
| **P1H1*** | 95.47%  (94.96%,95.99%) | | |
| **P1H2** | 94.61%  (94.05%,95.17%) | 94.67%  (94.12%,95.23%) | 94.69%  (94.13%,95.24%) |
| **P2H1** | 93.19%  (92.56%,93.81%) | 93.51%  (92.89%,94.12%) | 93.63%  (93.03%,94.24%) |
| **P2H2** | 91.19%  (90.48%,91.89%) | 91.81%  (91.13%,92.49%) | 92.05%  (91.38%,92.72%) |
| **P3H1** | 90.99%  (90.28%,91.70%) | 91.70%  (91.01%,92.38%) | 91.92%  (91.25%,92.60%) |
| **P3H2** | 87.68%  (86.87%,88.50%) | 89.01%  (88.23%,89.79%) | 89.51%  (88.75%,90.27%) |
| **P4H1** | 89.20%  (88.43%,89.97%) | 90.26%  (89.52%,90.99%) | 90.69%  (89.97%,91.41%) |
| **P4H2** | 84.80%  (83.91%,85.69%) | 86.77%  (85.93%,87.61%) | 87.59%  (86.77%,88.40%) |
| **P5H1** | 86.79%  (85.95%,87.63%) | 88.87%  (88.09%,89.65%) | 89.39%  (88.63%,90.16%) |
| **P5H2** | 80.82%  (79.84%,81.80%) | 84.61%  (83.72%,85.50%) | 85.68%  (84.81%,86.55%) |

*The H1 and P1 events were unbounded by search windows since they contained single events; hence, the algorithm P1H1 referred to 1 HIV-related healthcare practitioner, or 1 HIV-related hospitalization occurring at any time.

**C-statistic and Kappa**

The area-under-the-curve (AUC) – also known as the Concordance-/C-statistic (43): When comparing binary measures with a binary reference standard: $AUC= \frac{Sensitivity+Specificity}{2}$. A value of 1 indicates perfect classification of ‘true’ HIV status, whereas a value of 0.5 indicates classification accuracy no better than random chance. The Kappa statistic measures the extent of absolute agreement between the algorithm-identified case status and the laboratory-confirmed status, defined as $\frac{(P_{o} - P_{e})}{(1-P_{e})}$ where *P_o_* is the proportion of observed agreement, and *P_e_* is the proportion of chance-expected agreement (44). A value of 1 indicates absolute agreement between the algorithm-identified and laboratory-confirmed HIV status.

C-statistic values ranged from 90.35% (95% CI: 89.86%, 90.85%) for the five HIV-related healthcare practitioner encounters or two hospitalizations within a 12-month window algorithm to 96.31% (95% CI: 95.96% - 96.67%) for the two HIV-related healthcare practitioner encounters within a 36-month window, or one HIV-related hospitalization ever algorithm. Kappa ranged from 71.60% (95% CI: 70.17% - 73.04%) for the 5 HIV-related healthcare practitioner encounters or two hospitalizations within a 12-month window algorithm to 90.62% (95% CI: 89.67% - 91.57%) for the one HIV-related healthcare practitioner encounter or one hospitalization ever algorithm. Higher values on both composite measures (indicating better concordance and agreement between HIV status as per the algorithm and the HIV test classifications) tended to be among the most sensitive algorithms – requiring one or two HIV-related healthcare practitioner encounters. The reverse pattern was also true – algorithms with the lowest sensitivity (and highest specificity) tended to have the lowest C-statistic and lowest Kappa. C-statistics equally weight sensitivity and specificity, and since specificity was generally ‘high’ with little variability (values ranging from approximately 97% to 100%) yet sensitivity varied much more (from 80% to 9%) – variation in C-statistics was mostly a function of variation in sensitivity.

**Table S7. C-statistic estimates (95% confidence intervals), stratified by search window**

|  | **Search window length (time-window in which HIV-related healthcare events must co-occur)** | | |
| --- | --- | --- | --- |
|  | **12-month** | **24-month** | **36-month** |
| **P1H1*** | 96.30%  (95.89%,96.71%) | | |
| **P1H2** | 96.04%  (95.63%,96.45%) | 96.07%  (95.66%,96.48%) | 96.08%  (95.67%,96.48%) |
| **P2H1** | 96.09%  (95.72%,96.45%) | 96.25%  (95.89%,96.61%) | 96.31%  (95.96%,96.67%) |
| **P2H2** | 95.28%  (94.89%,95.66%) | 95.59%  (95.22%,95.96%) | 95.71%  (95.34%,96.08%) |
| **P3H1** | 95.18%  (94.79%,95.57%) | 95.53%  (95.16%,95.91%) | 95.64%  (95.27%,96.01%) |
| **P3H2** | 93.71%  (93.29%,94.13%) | 94.37%  (93.97%,94.77%) | 94.62%  (94.23%,95.01%) |
| **P4H1** | 94.36%  (93.95%,94.77%) | 94.87%  (94.48%,95.26%) | 95.08%  (94.70%,95.47%) |
| **P4H2** | 92.35%  (91.90%,92.79%) | 93.31%  (92.88%,93.74%) | 93.72%  (93.30%,94.13%) |
| **P5H1** | 93.15%  (92.71%,93.59%) | 94.17%  (93.76%,94.59%) | 94.44%  (94.03%,94.84%) |
| **P5H2** | 90.35%  (89.86%,90.85%) | 92.23%  (91.78%,92.68%) | 92.77%  (92.33%,93.21%) |

*The H1 and P1 events were unbounded by search window since they contained single events; hence, the algorithm P1H1 referred to 1 HIV-related healthcare practitioner, or 1 HIV-related hospitalization occurring at any time.

**Table S8. Kappa values (95% confidence intervals), stratified by search window**

|  | **Search window length (time-window in which HIV-related healthcare events must co-occur)** | | |
| --- | --- | --- | --- |
|  | **12-month** | **24-month** | **36-month** |
| **P1H1*** | 90.62%  (89.67%,91.57%) | | |
| **P1H2** | 89.52%  (88.52%,90.51%) | 89.61%  (88.62%,90.61%) | 89.64%  (88.65%,90.63%) |
| **P2H1** | 88.43%  (87.40%,89.46%) | 88.92%  (87.90%,89.93%) | 89.11%  (88.11%,90.12%) |
| **P2H2** | 85.69%  (84.56%,86.82%) | 86.62%  (85.52%,87.72%) | 86.98%  (85.89%,88.06%) |
| **P3H1** | 85.40%  (84.27%,86.54%) | 86.45%  (85.35%,87.55%) | 86.79%  (85.69%,87.88%) |
| **P3H2** | 80.86%  (79.60%,82.13%) | 82.77%  (81.55%,83.98%) | 83.49%  (82.29%,84.68%) |
| **P4H1** | 82.88%  (81.67%,84.10%) | 84.40%  (83.23%,85.57%) | 85.03%  (83.88%,86.18%) |
| **P4H2** | 76.94%  (75.60%,78.29%) | 79.65%  (78.36%,80.94%) | 80.81%  (79.54%,82.07%) |
| **P5H1** | 79.44%  (78.14%,80.73%) | 82.37%  (81.15%,83.60%) | 83.14%  (81.93%,84.34%) |
| **P5H2** | 71.60%  (70.17%,73.04%) | 76.66%  (75.30%,78.01%) | 78.13%  (76.81%,79.46%) |

*The H1 and P1 events were unbounded by search windows since they contained single events; hence, the algorithm P1H1 referred to 1 HIV-related healthcare practitioner, or 1 HIV-related hospitalization occurring at any time.

**Stratification of HIV case-finding algorithm results by sex***

**Figure S3. Stratification of sensitivity results, by sex**

**
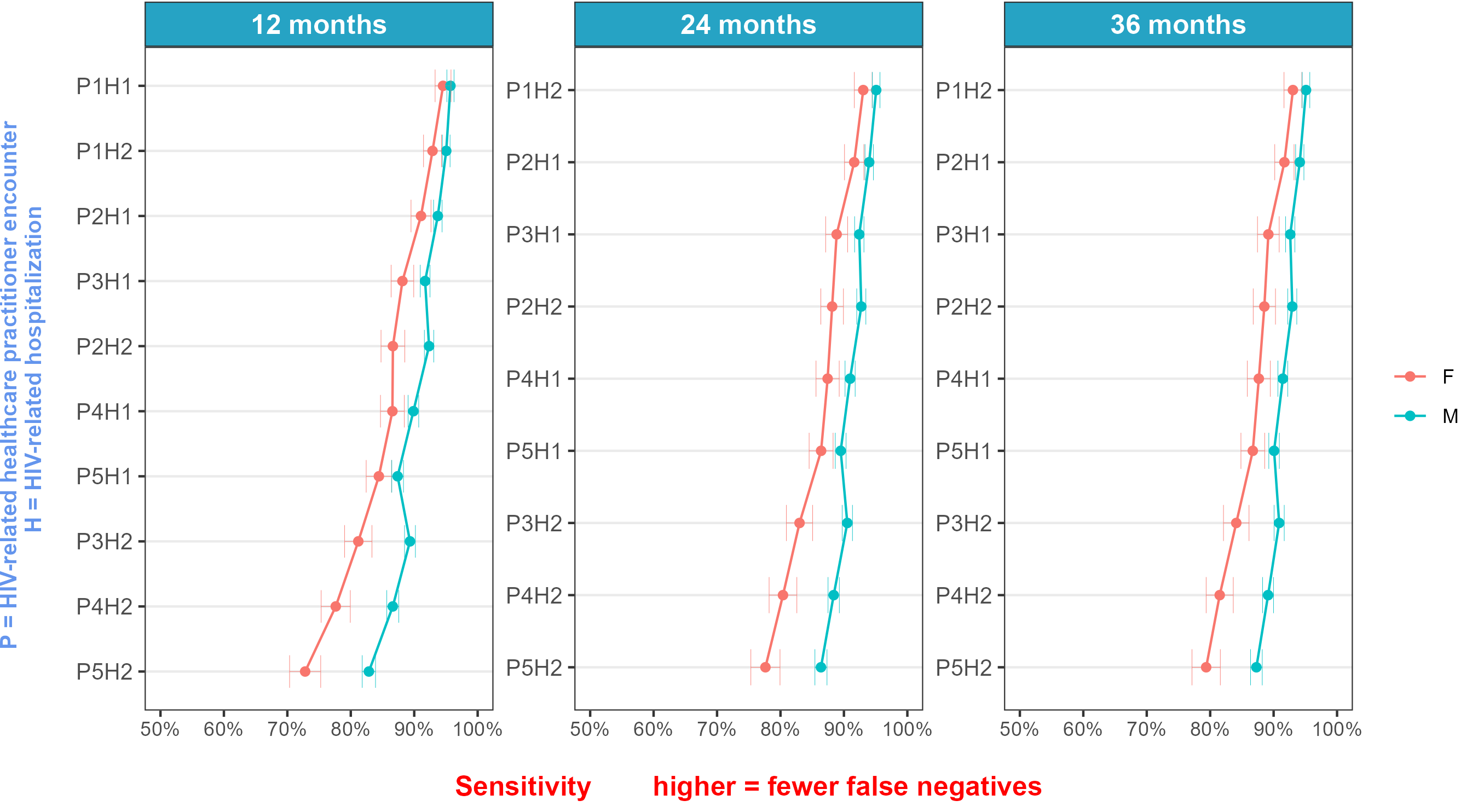
**

* The sex variable was based on the information collected at the time of registering for MSP (BC’s universal, public health insurance program), as it appears in the Client Registry Form. Although the field is termed ‘Gender’ on the MSP registration form, only the options ‘M’ and ‘F’ are available. Hence, from this variable, it was not possible to distinguish sex at birth, legal sex, and gender. The H1 and P1 events were unbounded by search windows since they contained single events; hence, the algorithm P1H1 referred to 1 HIV-related healthcare practitioner, or 1 HIV-related hospitalization occurring at any time.

**Figure S4. Stratification of specificity results, by sex (Note: x-axis scale)**

**
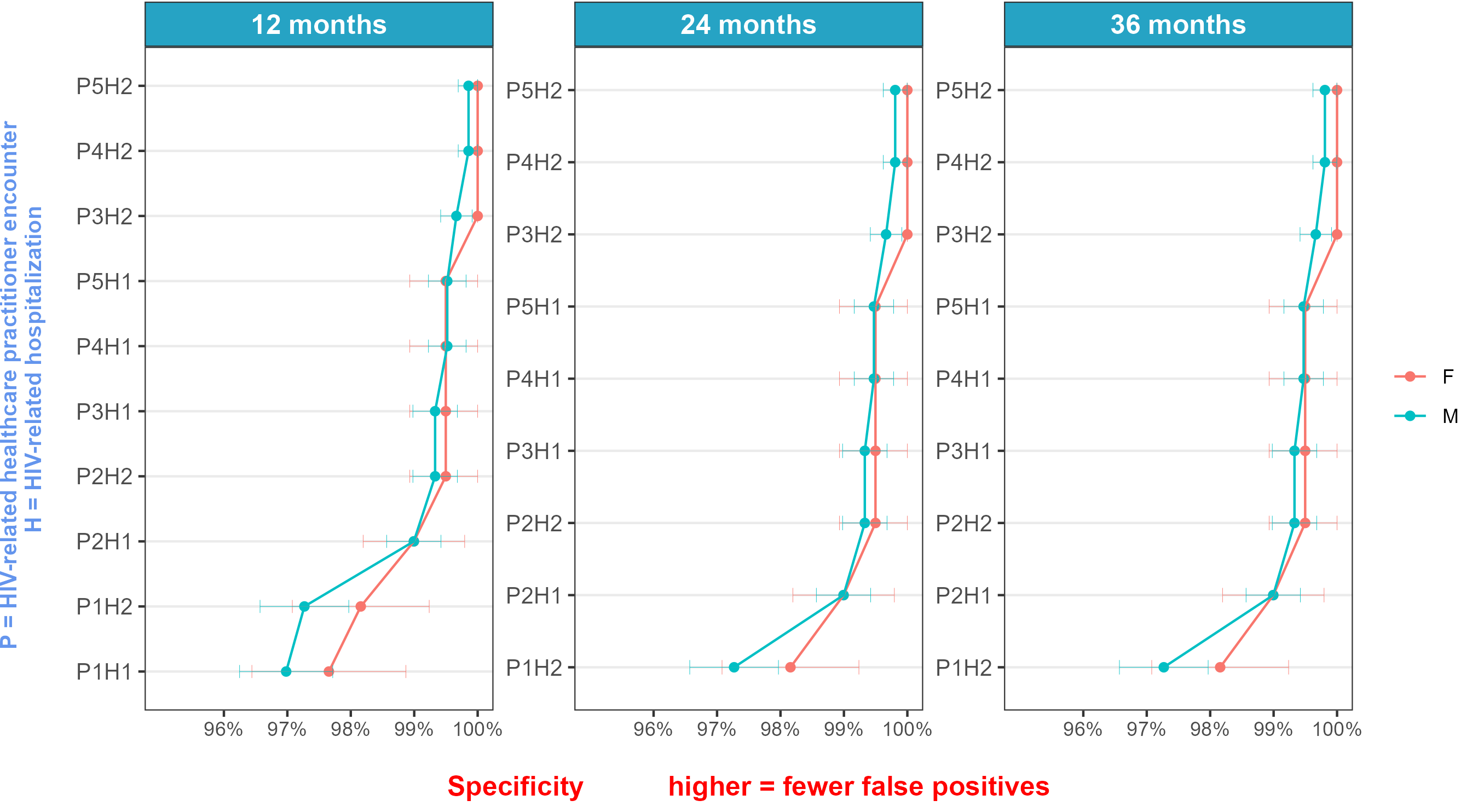
**

*The H1 and P1 events were unbounded by search windows since they contained single events; hence, the algorithm P1H1 referred to 1 HIV-related healthcare practitioner, or 1 HIV-related hospitalization occurring at any time.

**Stratification of HIV case-finding algorithm results by age (at earliest HIV-related record)**

**Figure S5. Stratification of sensitivity results, by age at earliest HIV-related record (HIV-related healthcare practitioner encounter, HIV-related hospitalization, or BC-CfE holdings)**


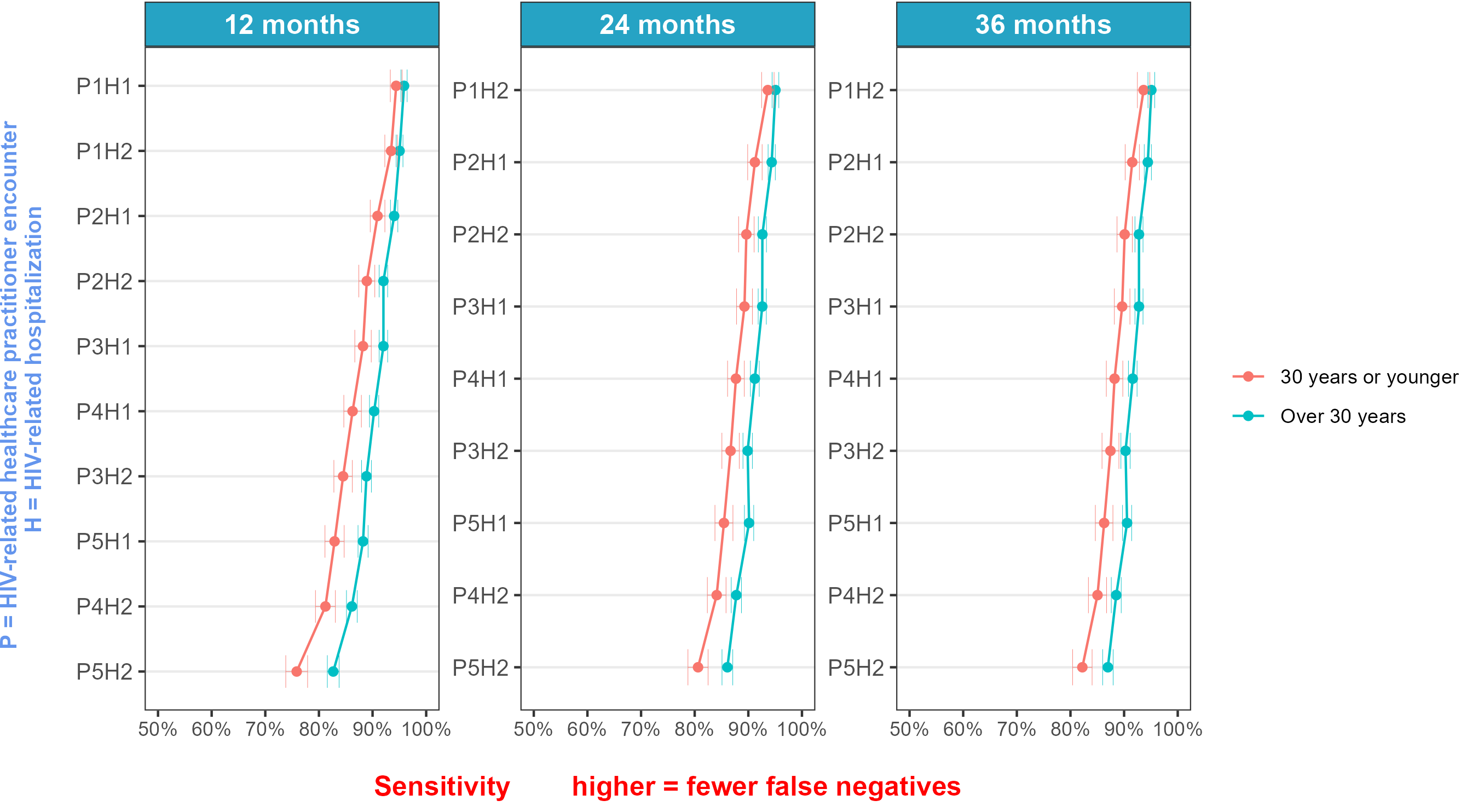


*The H1 and P1 events were unbounded by search windows since they contained single events; hence, the algorithm P1H1 referred to 1 HIV-related healthcare practitioner, or 1 HIV-related hospitalization occurring at any time.

**Figure S6. Stratification of specificity results, by age at earliest HIV-related record (HIV-related healthcare practitioner encounter, HIV-related hospitalization, or BC-CfE holdings)**

**
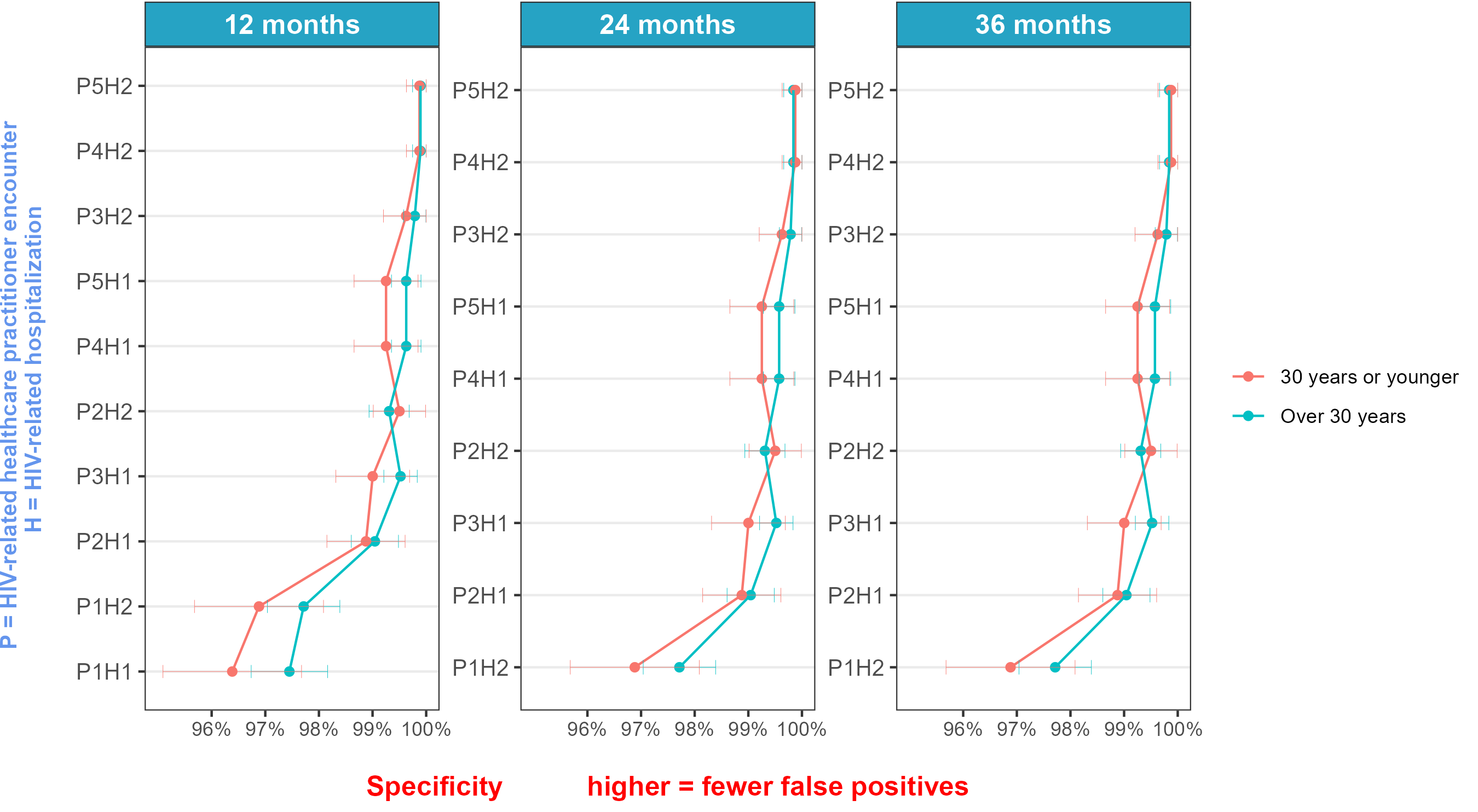
**

*The H1 and P1 events were unbounded by search windows since they contained single events; hence, the algorithm P1H1 referred to 1 HIV-related healthcare practitioner, or 1 HIV-related hospitalization occurring at any time.

**Stratification of HIV case-finding algorithm results by key population group**

**Figure S7. Stratification of sensitivity results, by MSM status**

**
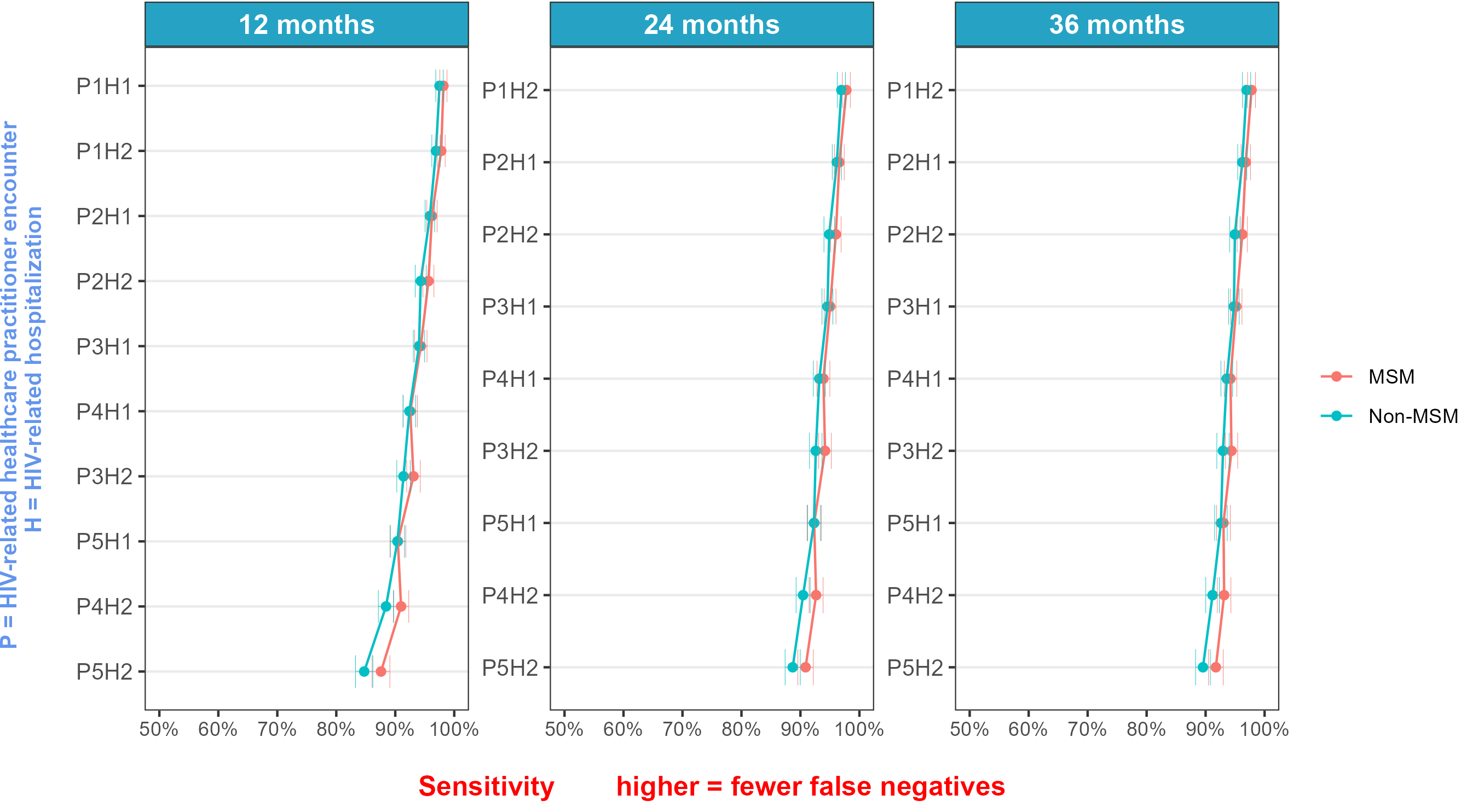
**

MSM: men who self-identified as men who have sex with men, based on BC-CfE records.*The H1 and P1 events were unbounded by search windows since they contained single events; hence, the algorithm P1H1 referred to 1 HIV-related healthcare practitioner, or 1 HIV-related hospitalization occurring at any time.

**Figure S8. Stratification of specificity results, by MSM status**


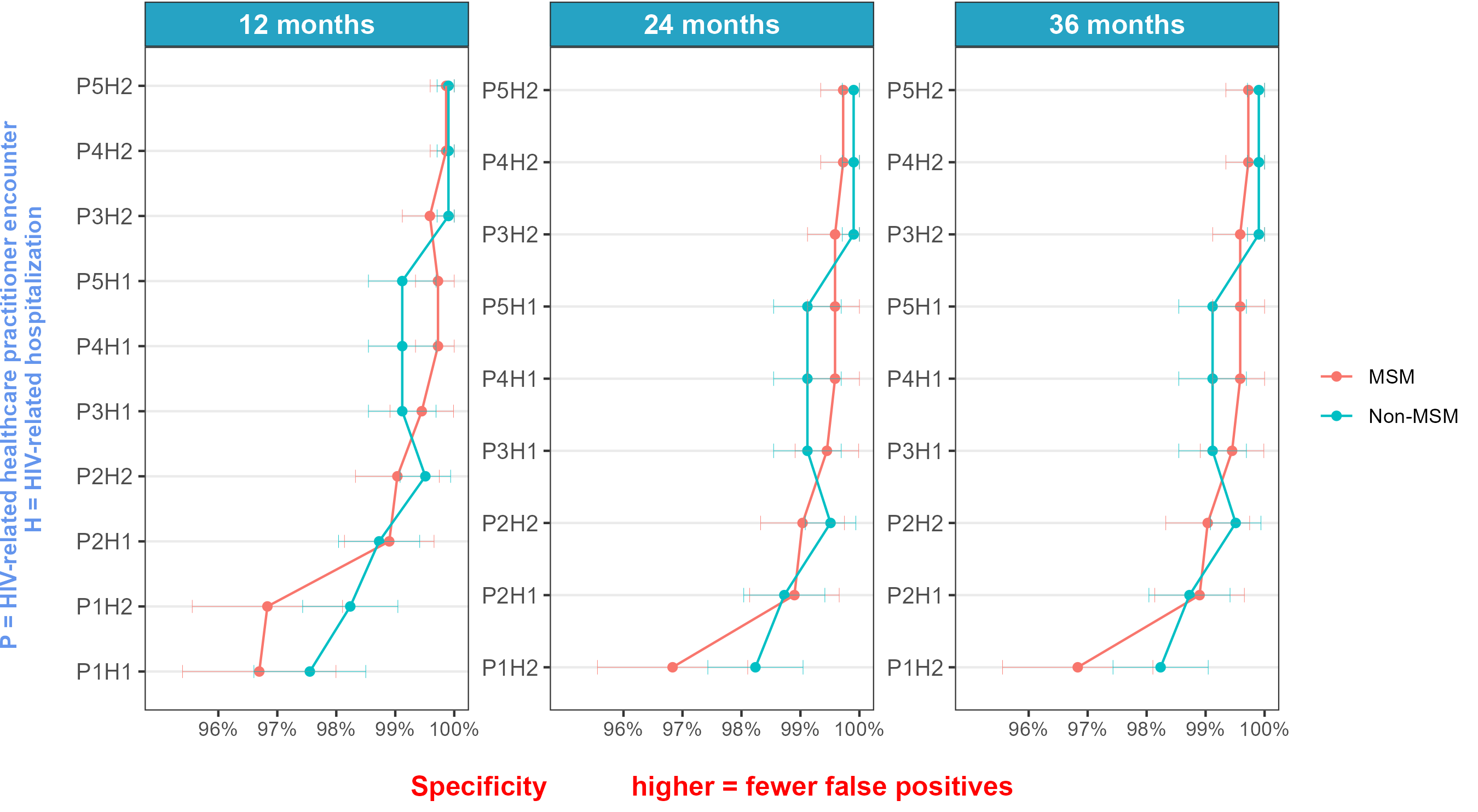


MSM: men who self identified as men who have sex with men, based on BC-CfE records. *The H1 and P1 events were unbounded by search windows since they contained single events; hence, the algorithm P1H1 referred to 1 HIV-related healthcare practitioner, or 1 HIV-related hospitalization occurring at any time.

**Figure S9. Stratification of sensitivity results, by IDU status**


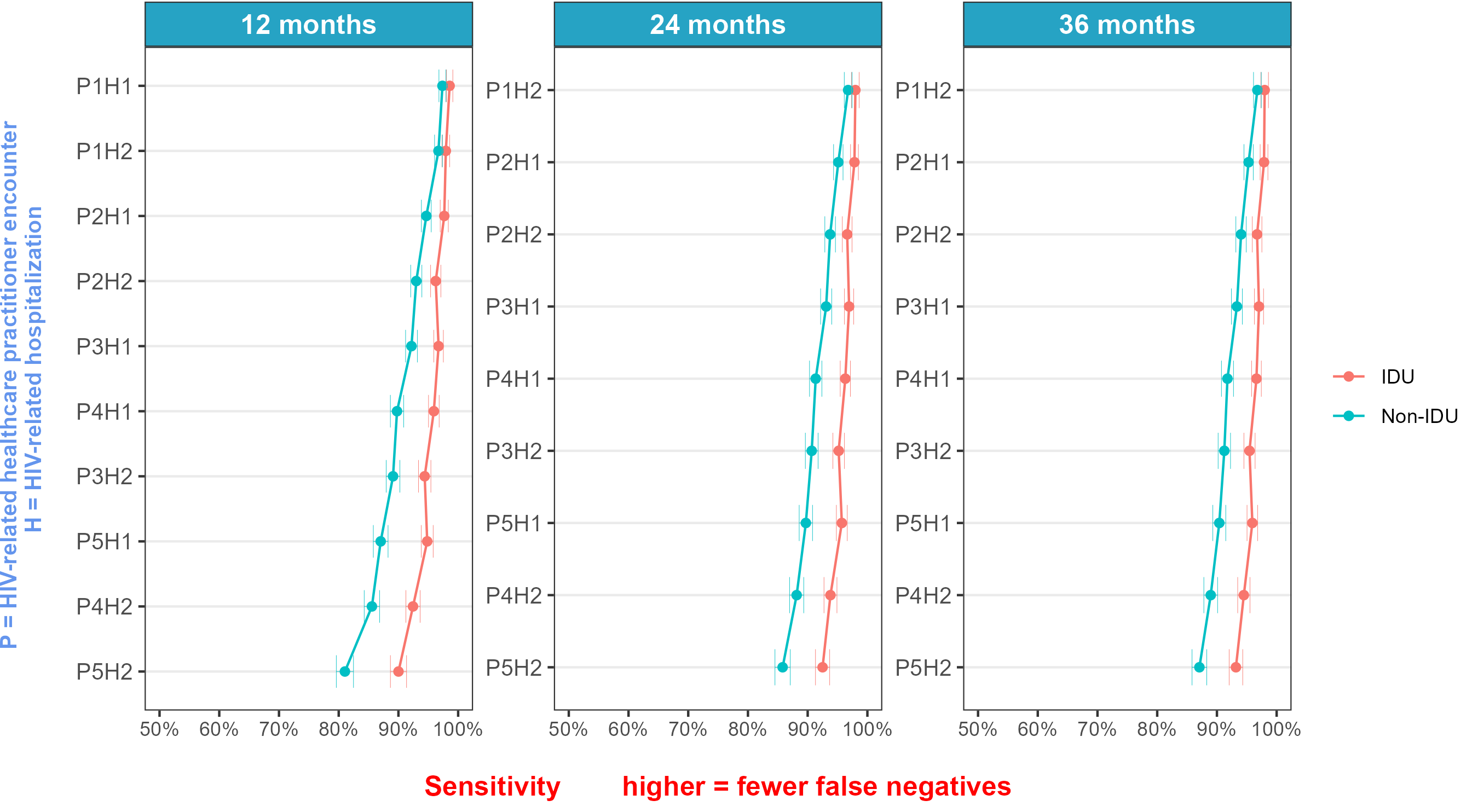


IDU: any self- or physician-reported history of injection drug use, based on BC-CfE records, BCCDC HIV test records (exposure category), or PharmaNet records (for medications for opioid agonist treatment). *The H1 and P1 events were unbounded by search windows since they contained single events; hence, the algorithm P1H1 referred to 1 HIV-related healthcare practitioner, or 1 HIV-related hospitalization occurring at any time.

**Figure S10. Stratification of specificity results, by IDU status**


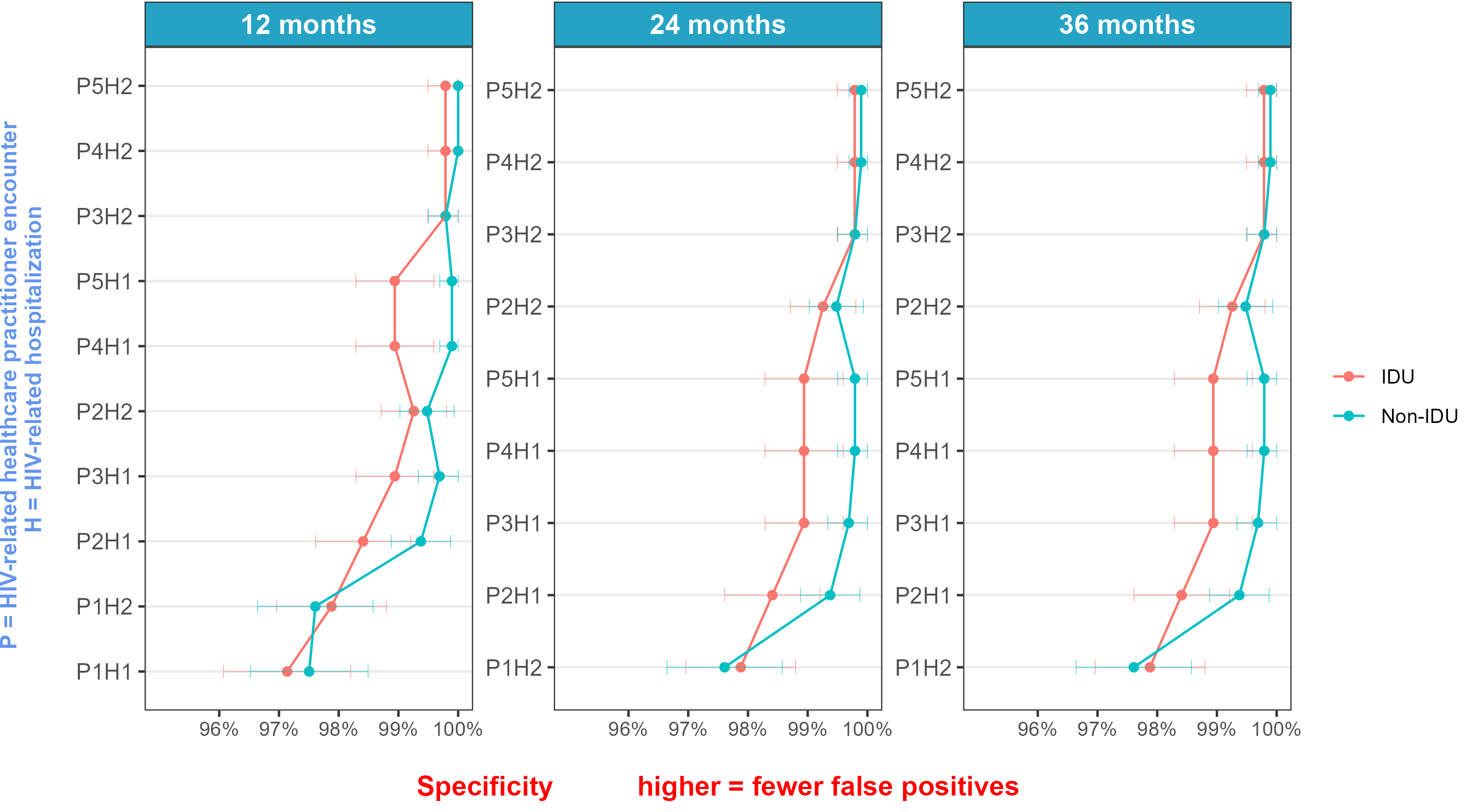


IDU: any self- or physician-reported history of injection drug use, based on BC-CfE records, BCCDC HIV test records (exposure category), or PharmaNet records (for medications for opioid agonist treatment). *The H1 and P1 events were unbounded by search windows since they contained single events; hence, the algorithm P1H1 referred to 1 HIV-related healthcare practitioner, or 1 HIV-related hospitalization occurring at any time.

**Figure S11. Stratification of sensitivity results, by Heterosexual status**


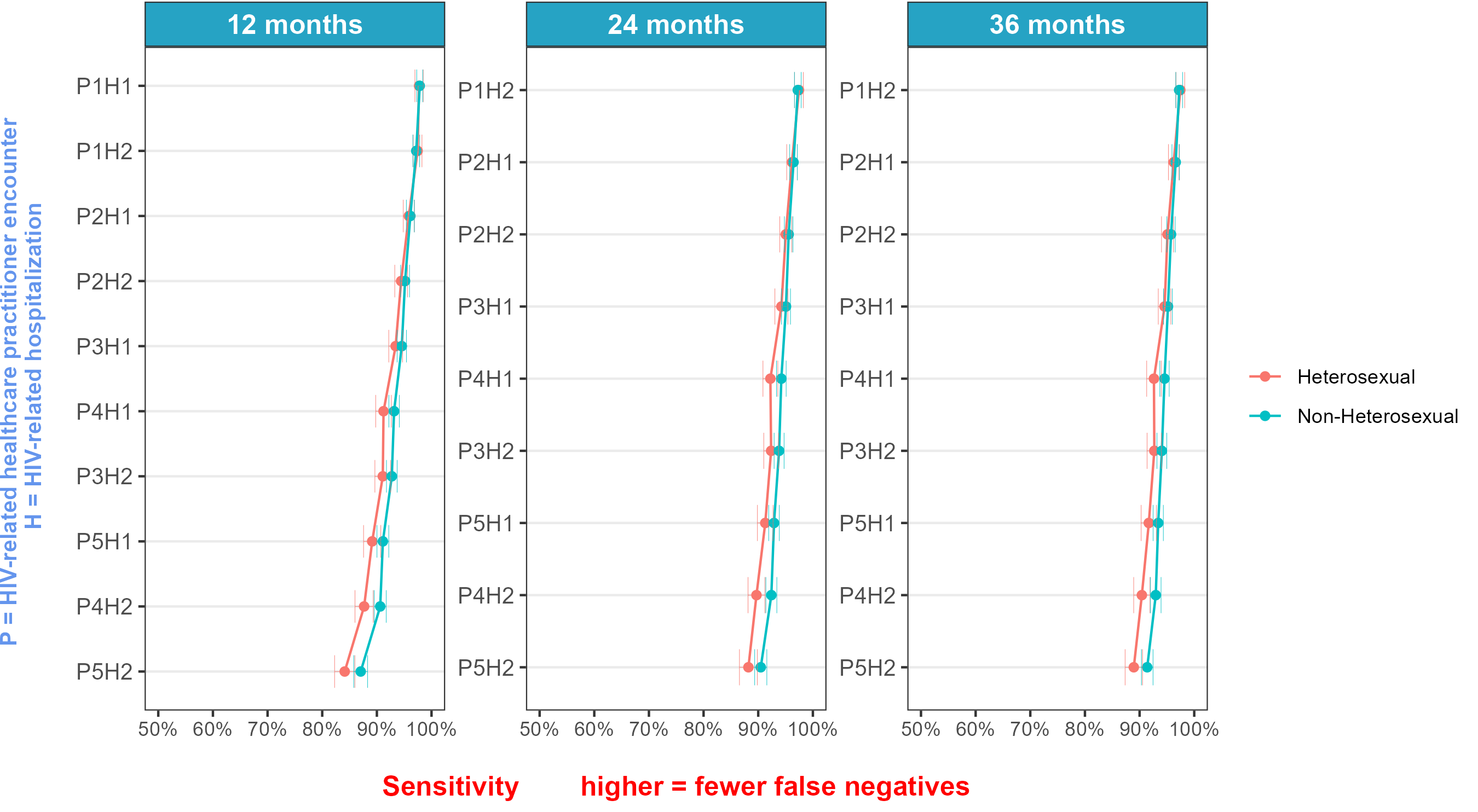


*The H1 and P1 events were unbounded by search windows since they contained single events; hence, the algorithm P1H1 referred to 1 HIV-related healthcare practitioner, or 1 HIV-related hospitalization occurring at any time.

**Figure S12. Stratification of specificity results, by Heterosexual status**


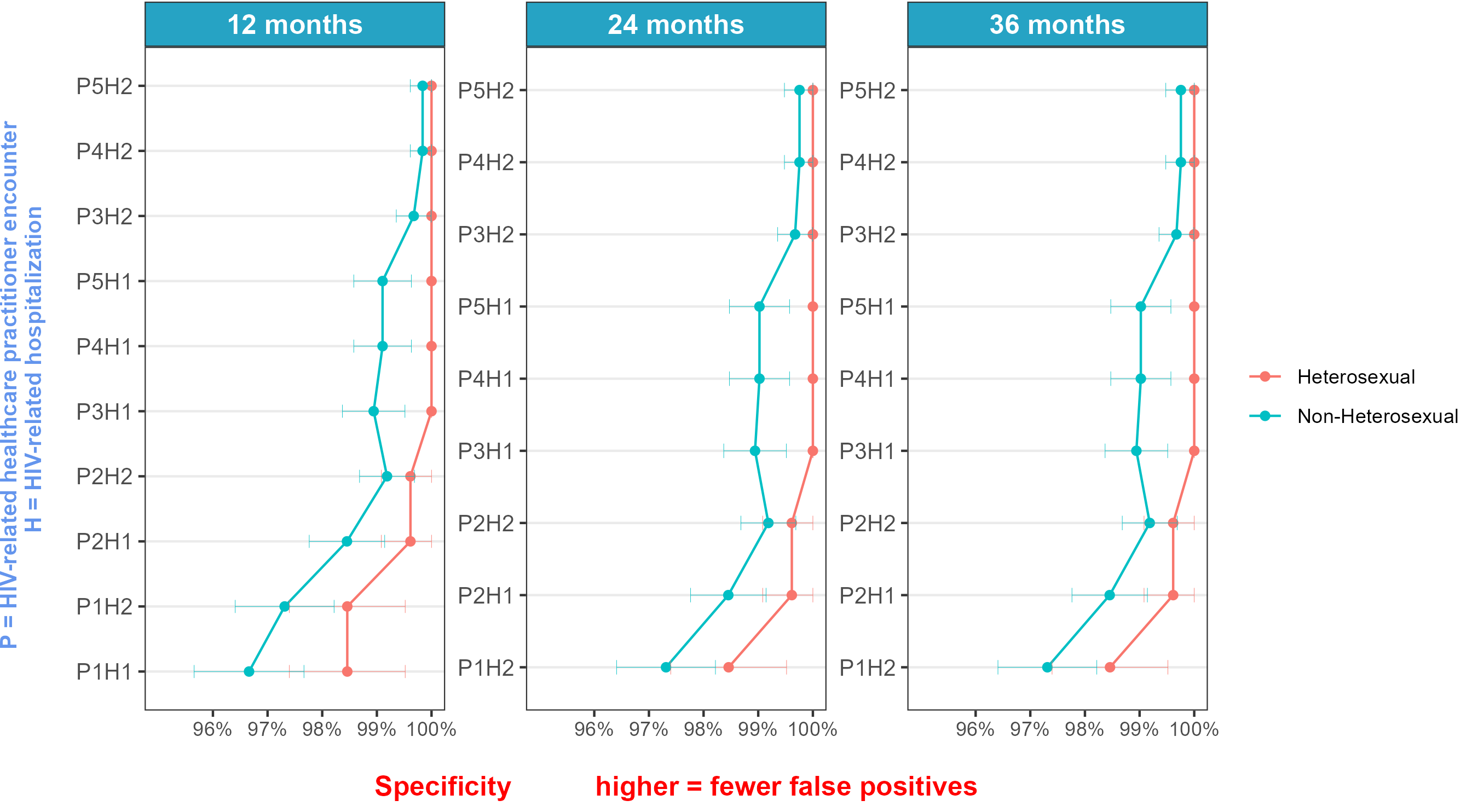


*The H1 and P1 events were unbounded by search windows since they contained single events; hence, the algorithm P1H1 referred to 1 HIV-related healthcare practitioner, or 1 HIV-related hospitalization occurring at any time.

**Estimated counts and characteristics of people with HIV in BC using algorithms**

**Table S9. Estimated count of people with HIV present and alive in BC as of March 2020, by algorithm used to supplement confirmed HIV case count from BC-CfE held records (i.e., HIV status indicated by detectable plasma viral load, ARV dispensation, or HIV/AIDS related death)**

|  | **Search window length (time-window in which HIV-related healthcare events must co-occur)** | | |
| --- | --- | --- | --- |
|  | **12-month** | **24-month** | **36-month** |
| **P1H1*** | 30,071 | | |
| **P1H2** | 29,776 | 29,780 | 29,784 |
| **P2H1** | 10,670 | 11,018 | 11,223 |
| **P2H2** | 10,340 | 10,694 | 10,903 |
| **P3H1** | 9,440 | 9,513 | 9,584 |
| **P3H2** | 9,101 | 9,178 | 9,253 |
| **P4H1** | 9,189 | 9,231 | 9,250 |
| **P4H2** | 8,842 | 8,889 | 8,913 |
| **P5H1** | 9,104 | 9,125 | 9,141 |
| **P5H2** | 8,755 | 8,781 | 8,802 |

*The H1 and P1 events were unbounded by search windows since they contained single events; hence, the algorithm P1H1 referred to 1 HIV-related healthcare practitioner, or 1 HIV-related hospitalization occurring at any time.

**Table S9: Characteristics of people with HIV (PWH) in BC, present as of March 2020 (n=8,774), stratified by means of identification**

|  | Algorithm identified (n=333) | Non-algorithm identified (n=8,441) |
| --- | --- | --- |
| Male | 66.7% (n=222) | 82.5% (n=6,966) |
| Median (Q1, Q3) age at earliest HIV-related record | 38 (30, 48) | 36 (30, 44) |
| Time period of earliest HIV-related record  Before March 2001  April 2001 – March 2006  April 2006 – March 2011  April 2011 – March 2016  April 2016 – March 2020 | 15.0% (n=50)  14.1% (n=47)  20.7% (n=69)  19.5% (n=65)  30.6% (n=102) | 33.5% (n=2,831)  17.4% (n=1,470)  17.2% (n=1,449)  17.4% (n=1,467)  14.5% (n=1,224) |
| VCHA resident at time of earliest HIV-related record | 38.4% (n=126) | 52.5% (n=4,048) |
| Median (Q1, Q3) years of healthcare records after earliest HIV-related record | 8.91 (3.07, 15.32) | 14.25 (6.91, 21.72) |
| Median (Q1, Q3) number of all-cause outpatient healthcare practitioner encounters per year | 6.90 (3.99, 11.34) | 7.83 (5.84, 10.08) |
| Median (Q1, Q3) number of HIV-related healthcare practitioner encounters per year | 0.66 (0.34, 1.05) | 2.31 (1.27, 3.34) |

*Q1: First quartile; Q3: Third quartile; VCHA: Vancouver Coastal Health Authority. Algorithm identified = PWH identified solely by meeting the criteria of the chosen algorithm (i.e., recorded 5 HIV-related healthcare practitioner encounters or 2 HIV-related hospitalizations within 1 year, or ever had a hospitalization with HIV listed as the most responsible diagnosis). Non-algorithm identified = PWH identified by having an antiretroviral medication, a detectable plasma viral load for HIV, and/or a positive HIV test reported to the BC Centre for Disease Control.

**Table S10: Characteristics of people with HIV (PWH) in BC, ever present 1996 to 2020 (n=15,957), stratified by means of identification**

|  | Algorithm identified (n=985) | Non-algorithm identified (n=14,972) |
| --- | --- | --- |
| Male | 61.6% (n=607) | 82.2% (n=12,300) |
| Median (Q1, Q3) age at earliest HIV-related record | 39 (31, 50) | 37 (30, 44) |
| Time period of earliest HIV-related record  Before March 2001  April 2001 – March 2006  April 2006 – March 2011  April 2011 – March 2016  April 2016 – March 2020 | 29.0% (n=286)  20.9% (n=206)  20.8% (n=205)  16.9% (n=166)  12.4% (n=122) | 45.1% (n=6,756)  17.8% (n=2,671)  14.7% (n=2,205)  12.9% (n=1,935)  9.4% (n=1,405) |
| VCHA resident at time of earliest HIV-related record | 27.9% (n=232) | 53.1% (n=6,996) |
| Median (Q1, Q3) years of healthcare records after earliest HIV-related record | 4.50 (0.54, 10.85) | 9.70 (3.60, 17.65) |
| Median (Q1, Q3) number of all-cause outpatient healthcare practitioner encounters per year | 10.16 (9.64, 11.07) | 9.67 (9.34, 9.94) |
| Median (Q1, Q3) number of HIV-related healthcare practitioner encounters per year | 0.74 (0.47, 0.98) | 2.54 (1.52, 3.09) |
| Died during follow-up | 19.5% (n=192) | 28.9% (n=4,323) |
| Alive and in follow-up at end of study (March 2020) | 333 (33.8%) | 8,441 (56.4%) |

*Q1: First quartile; Q3: Third quartile; VCHA: Vancouver Coastal Health Authority. Algorithm identified = PWH identified solely by meeting the criteria of the chosen algorithm (i.e., recorded 5 HIV-related healthcare practitioner encounters or 2 HIV-related hospitalizations within 1 year, or ever had a hospitalization with HIV listed as the most responsible diagnosis). Non-algorithm identified = PWH identified by having an antiretroviral medication, a detectable plasma viral load for HIV, a positive HIV test reported to the BC Centre for Disease Control, and/or an HIV/AIDS related death recorded.

**Figure S12. Association between specificity estimates from validation sub-sample and estimated total count of people with HIV, by algorithm and search window length**


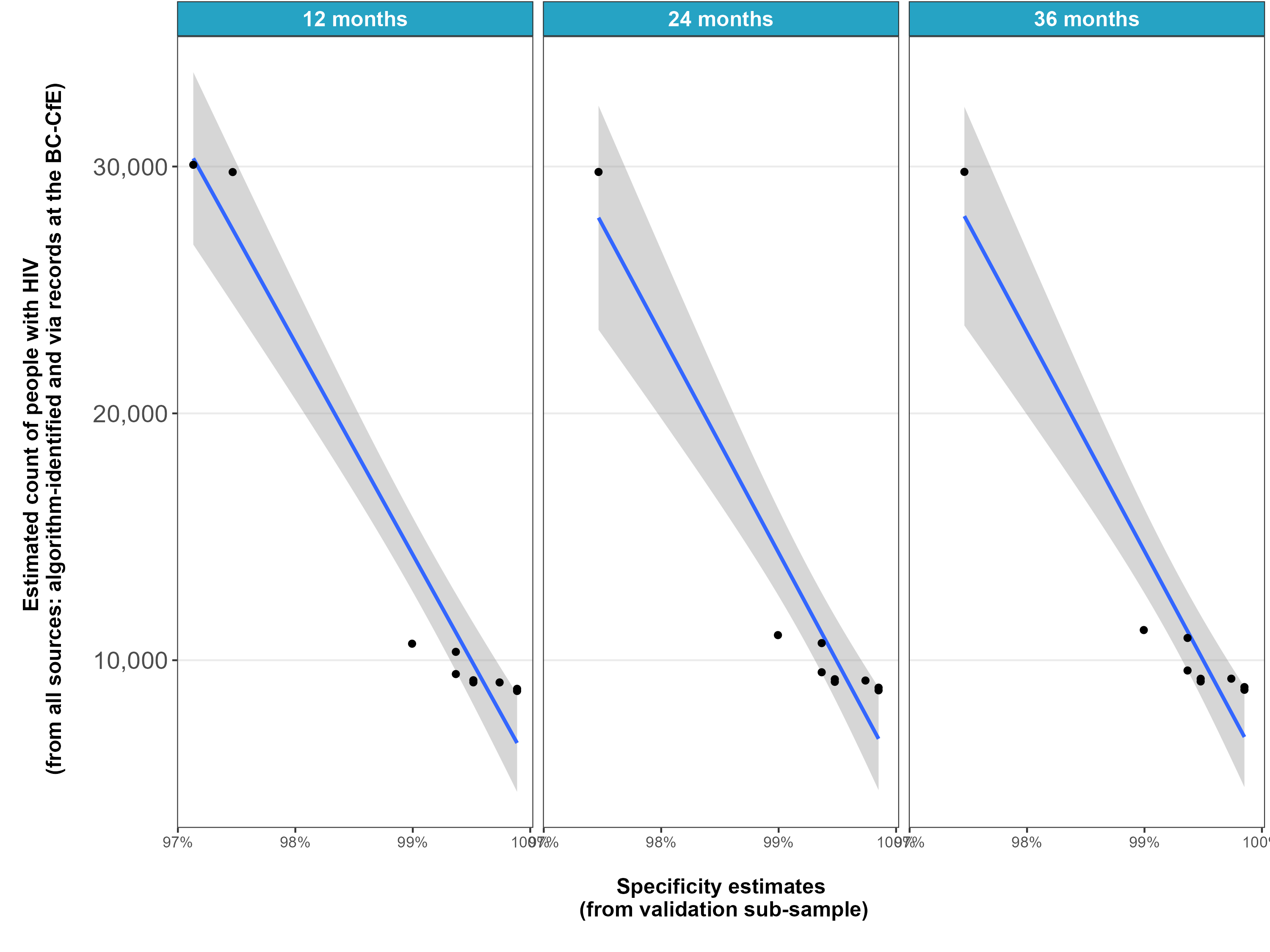


Excluding algorithms with a single healthcare practitioner encounter:


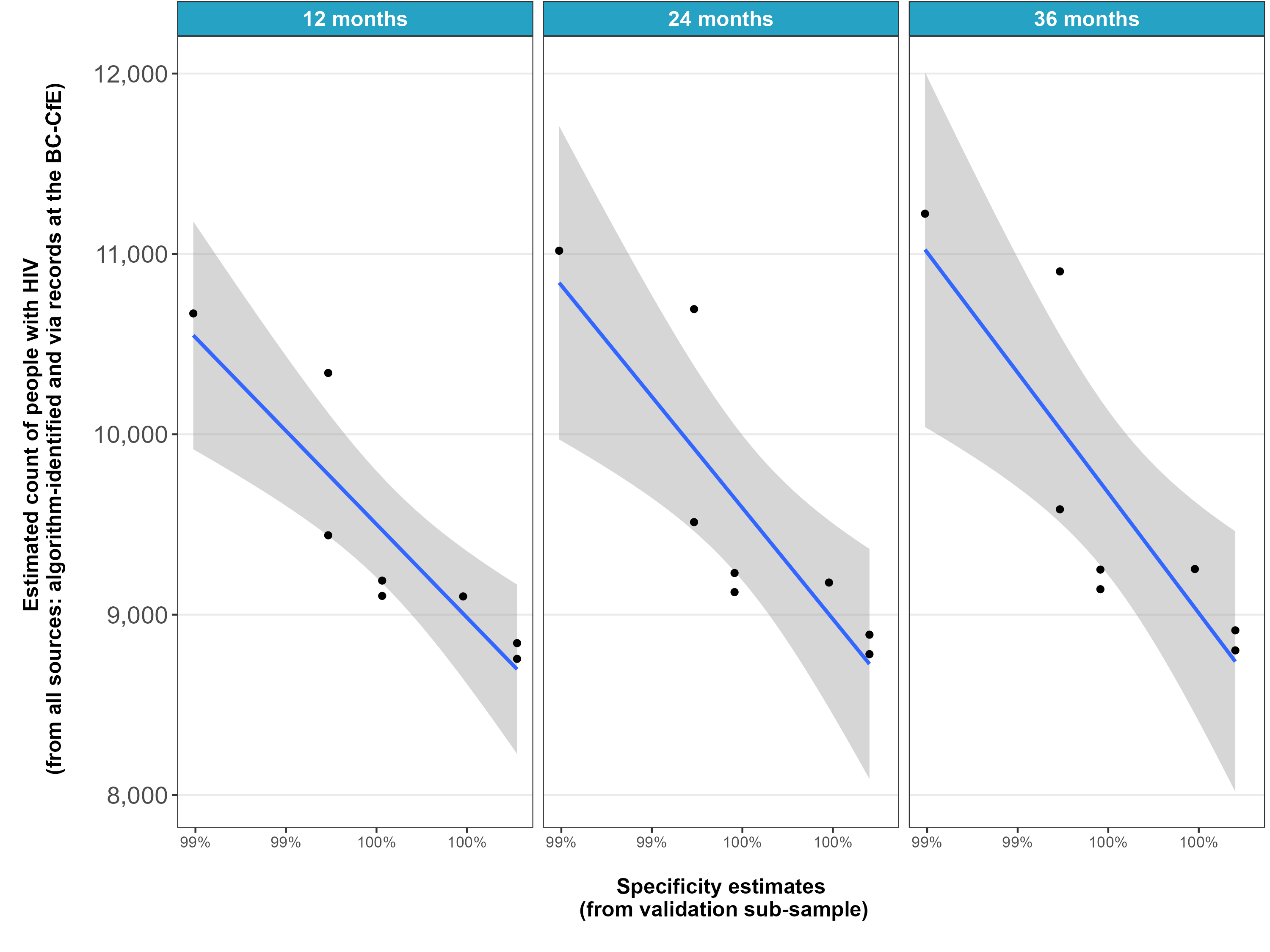

Supplement: S1 File — (DOCX) [file pone.0290777.s001.docx]
